# Supplementary material for: Transient rheology of the Sumatran mantle wedge revealed by a decade of great earthquakes
Source: Nat Commun. 2018 Mar 8;9:995. doi: 10.1038/s41467-018-03298-6 (PMC5843651; doi:10.1038/s41467-018-03298-6)
Supplement: Supplementary file 1 — Supplementary Information [file 41467_2018_3298_MOESM1_ESM.pdf]

# Transient rheology of the Sumatran mantle wedge revealed by a decade of great earthquakes

Qiu et al.

## Supplementary information

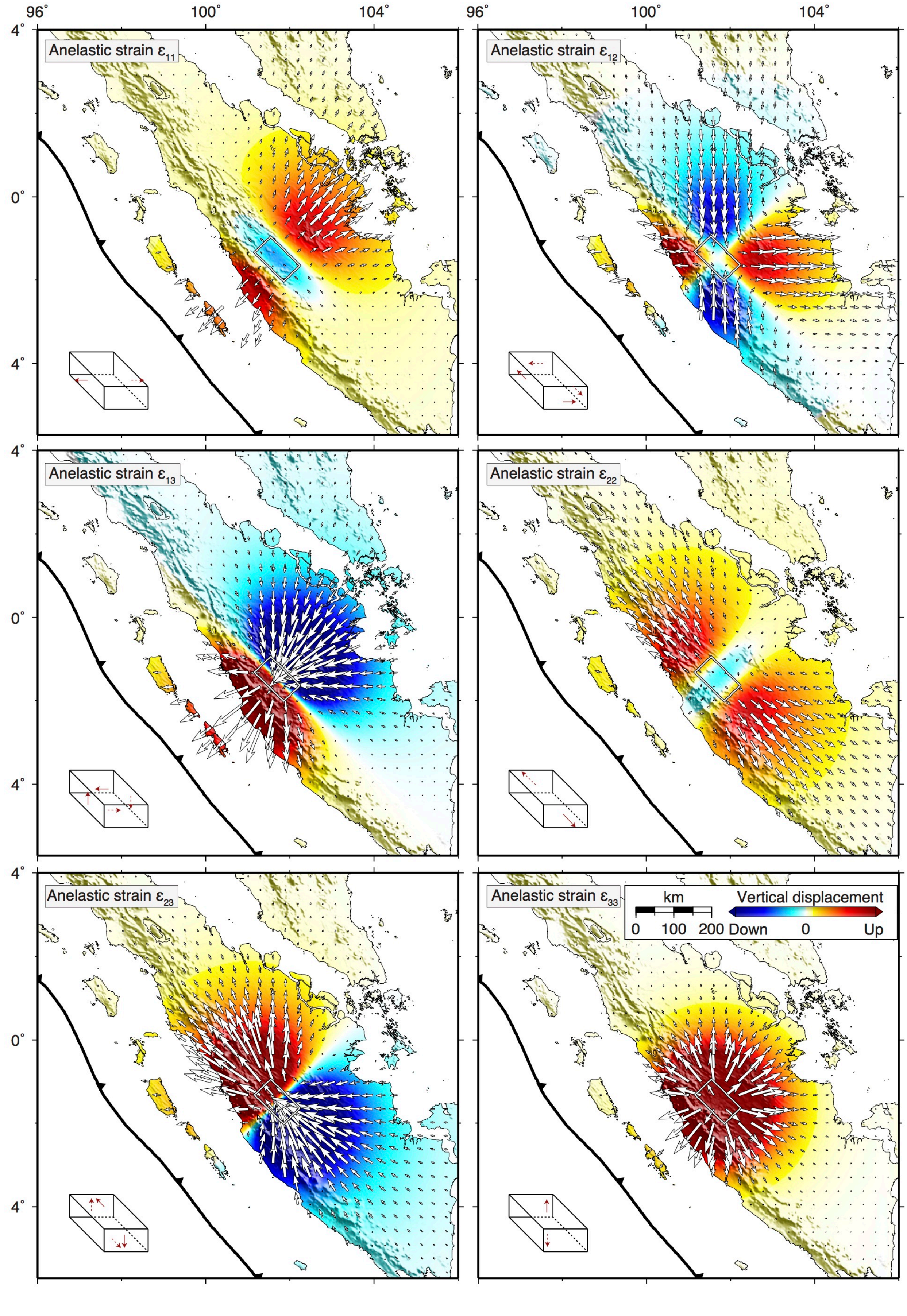


**Supplementary Figure 1. Surface displacements due to unit anelastic strain along six independent strain components within a buried deformable cuboid at a depth of ~100 km.** Vectors indicate horizontal displacements and colours represent vertical displacements. The surface projection of the example cuboid is shown as the white rectangle on each panel.

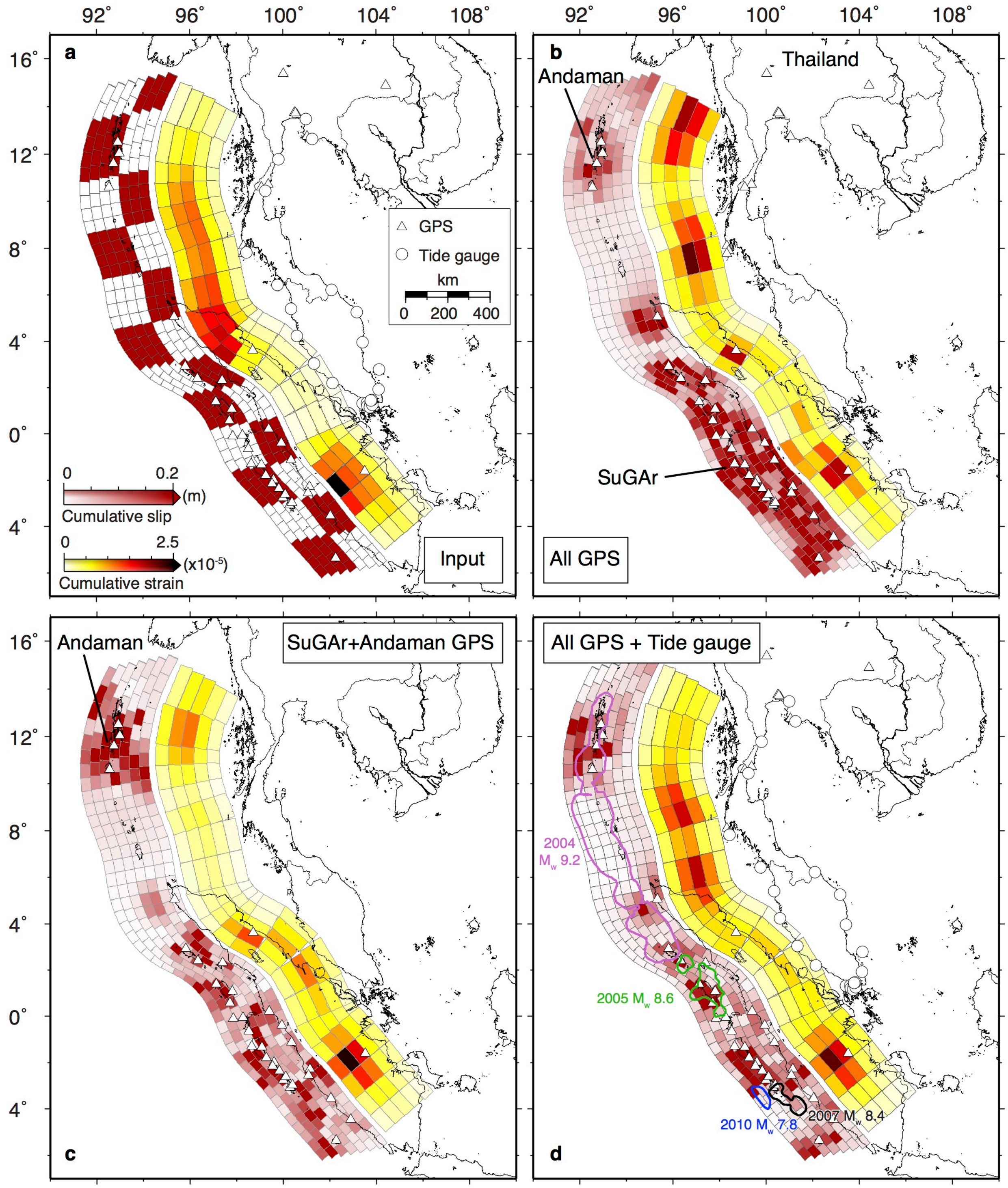


**Supplementary Figure 2. Checkerboard tests for slip and second invariant of strain tensor with different geodetic networks. a)** Checkerboard size ~220 km x ~180 km for slip, synthetic strain input for the inversion, and our near- and far-field networks; **b)** Inverted slip and strain with SuGAr GPS, Andaman and Thailand GPS; **c**) Inverted slip and strain with SuGAr GPS and Andaman GPS; **d**) Inverted slip and strain with all GPS and tide gauges.

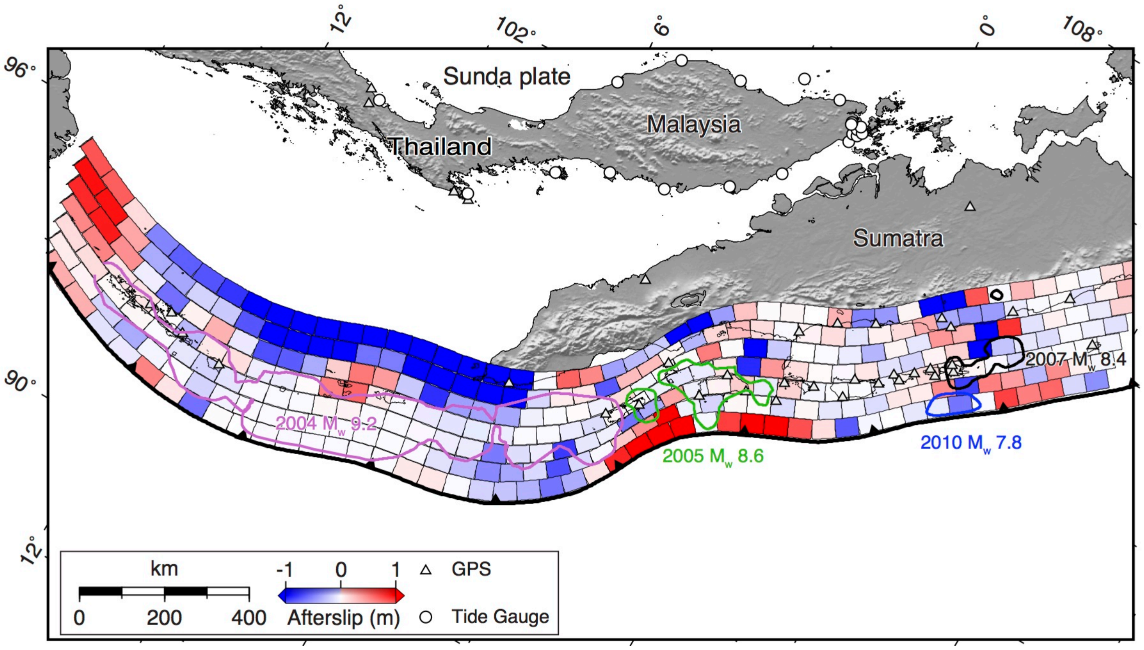


**Supplementary Figure 3. Differences in estimated cumulative afterslip (from early 2005 to 2014) between a model that includes viscous strain within the mantle wedge and one that does not. Slip contours and their corresponding time and magnitude are the same as for Fig. 1, 3 6 & 7.**

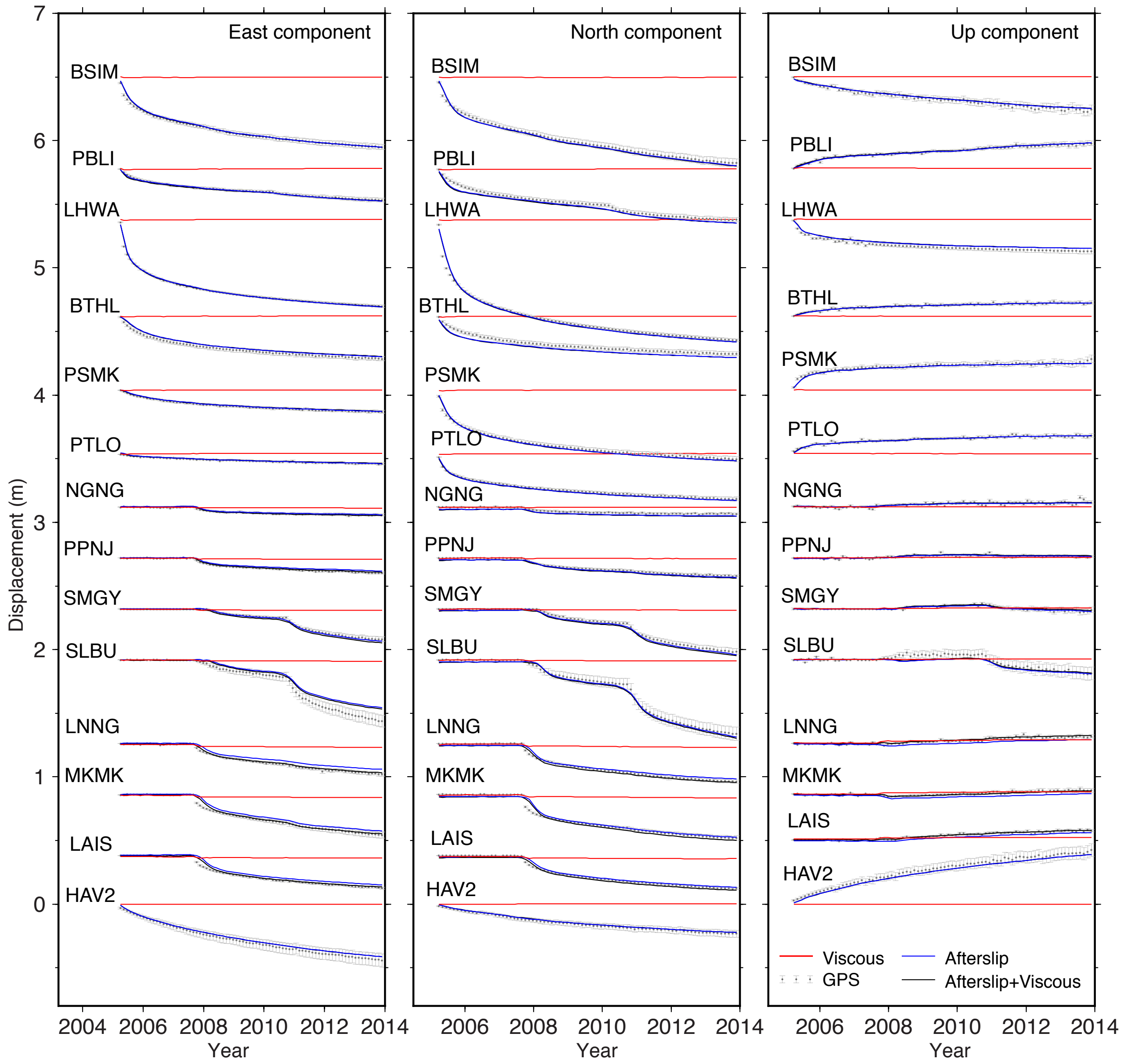


**Supplementary Figure 4. Best-fit model for all the GPS stations within our geodetic network.** The red, blue and black curves are model prediction from viscoelastic flow, afterslip and combination of both, respectively. GPS time series are plotted by a ~ 40 days interval for clarity and with 1σ error bars.

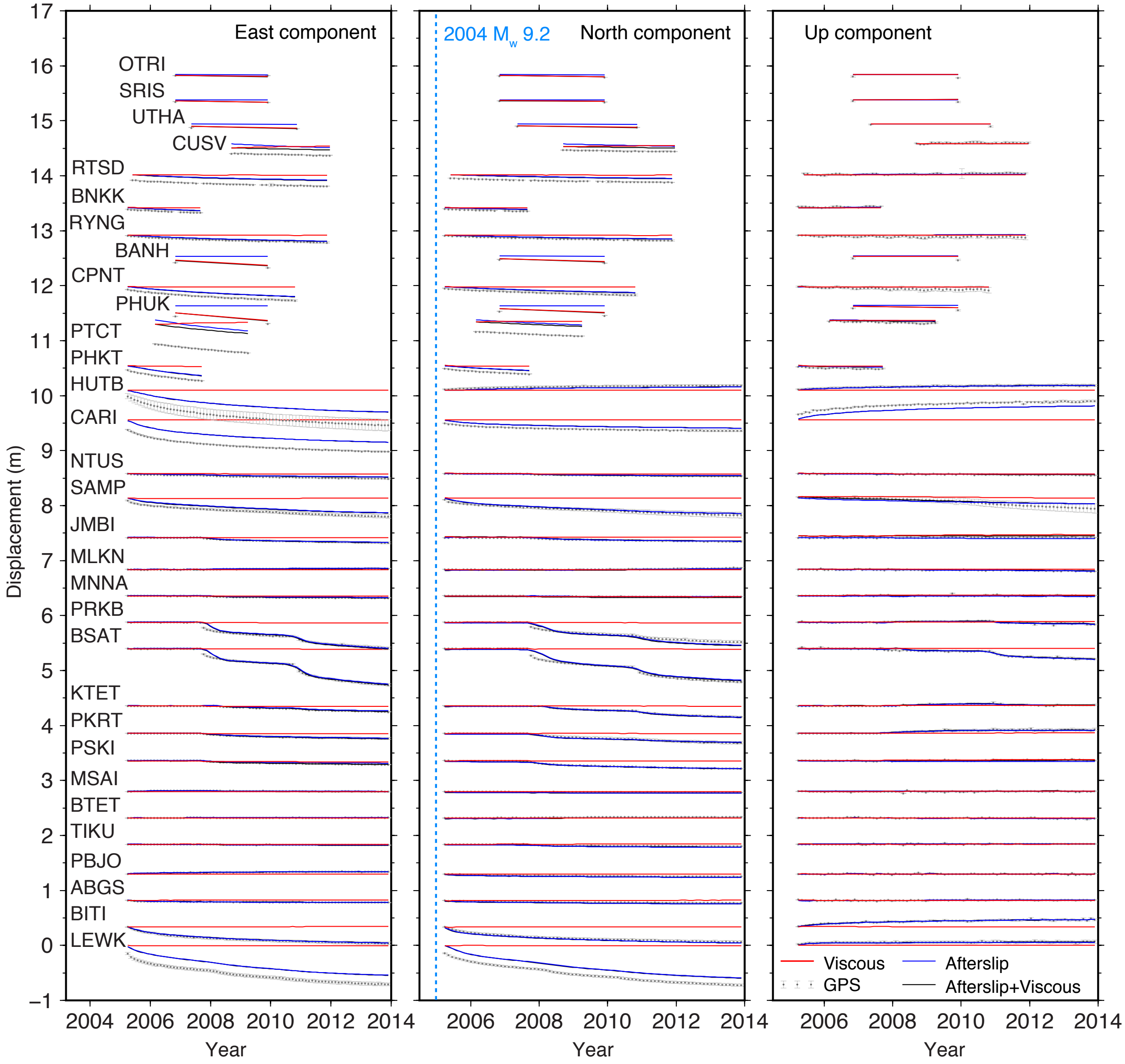


**Supplementary Figure 4. Continued.**

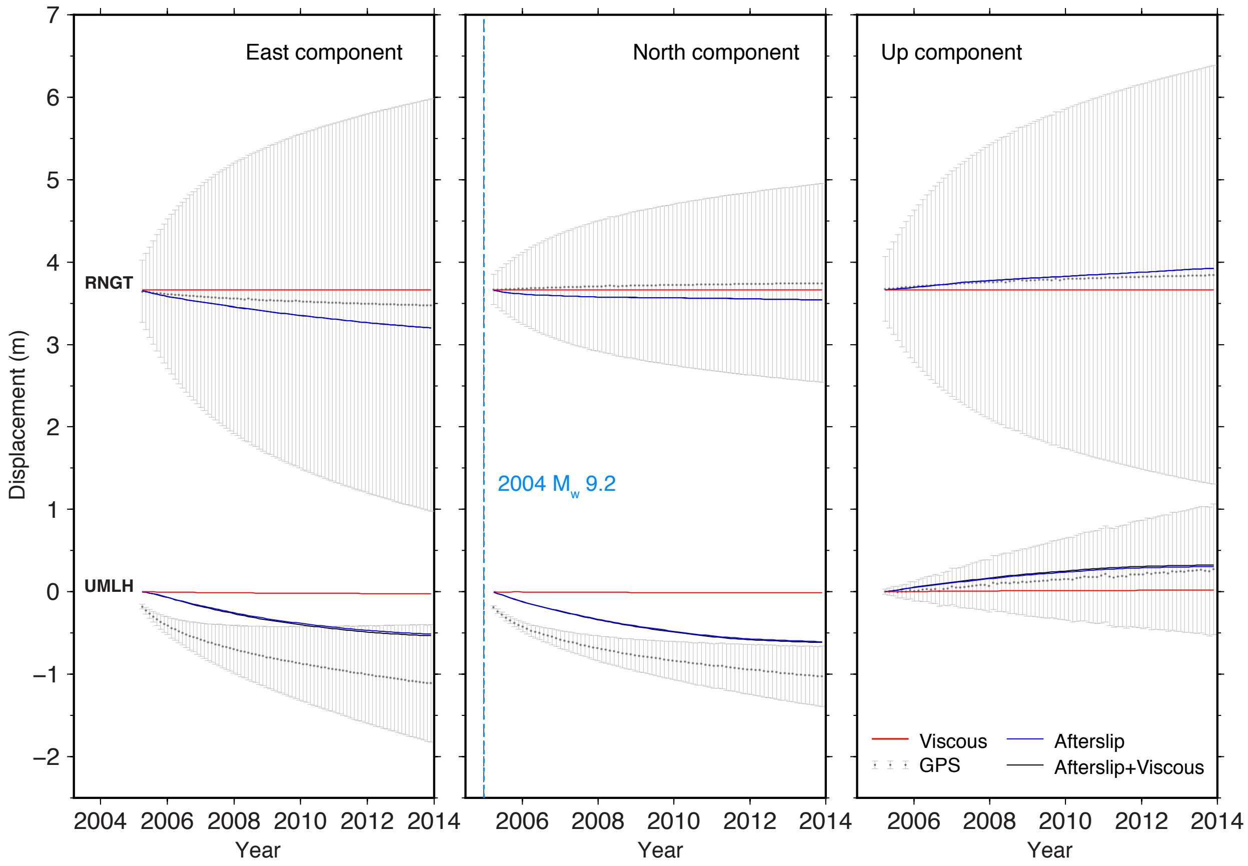


**Supplementary Figure 4. Continued.**

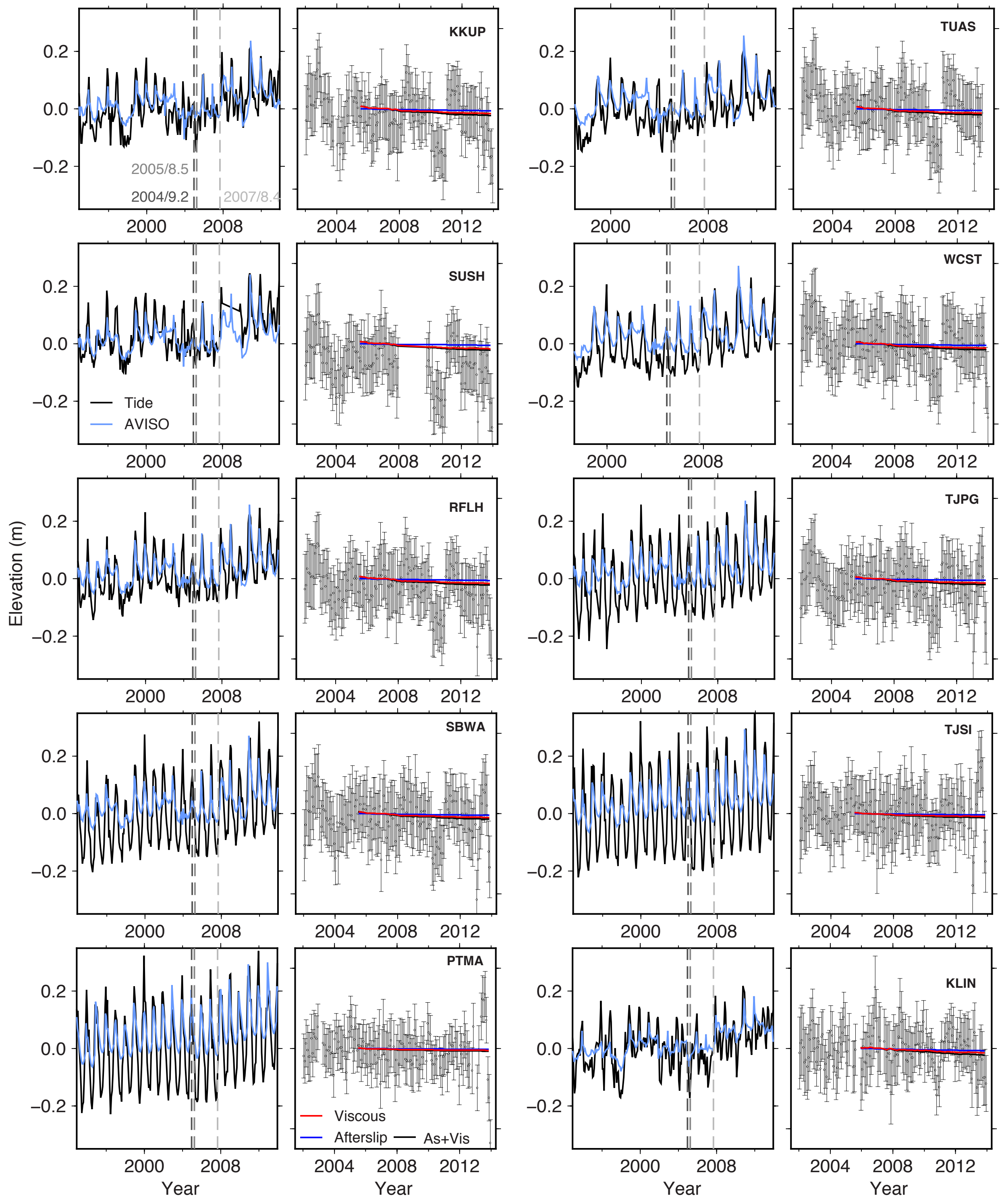


**Supplementary Figure 5. Best model predictions of vertical displacement fit with far-field tide gauge stations.** The red, blue and black curves are model prediction from viscoelastic flow, afterslip and combination of both, respectively. The first and third columns show the original tide and Aviso time series. The second and forth columns represent the derived land-height changes with 1σ error bars, where the red curves illustrate the mode predictions. The great megathrust earthquakes - the Mw 9.2 2004 Sumatra-Andaman, the Mw 8.5 2005 Nias-Simeulue, and the Mw 8.4 2007 Bengkulu earthquakes are indicated by the vertical dashed lines. The full name of the tide gauges are given in the legend of Fig. 2.

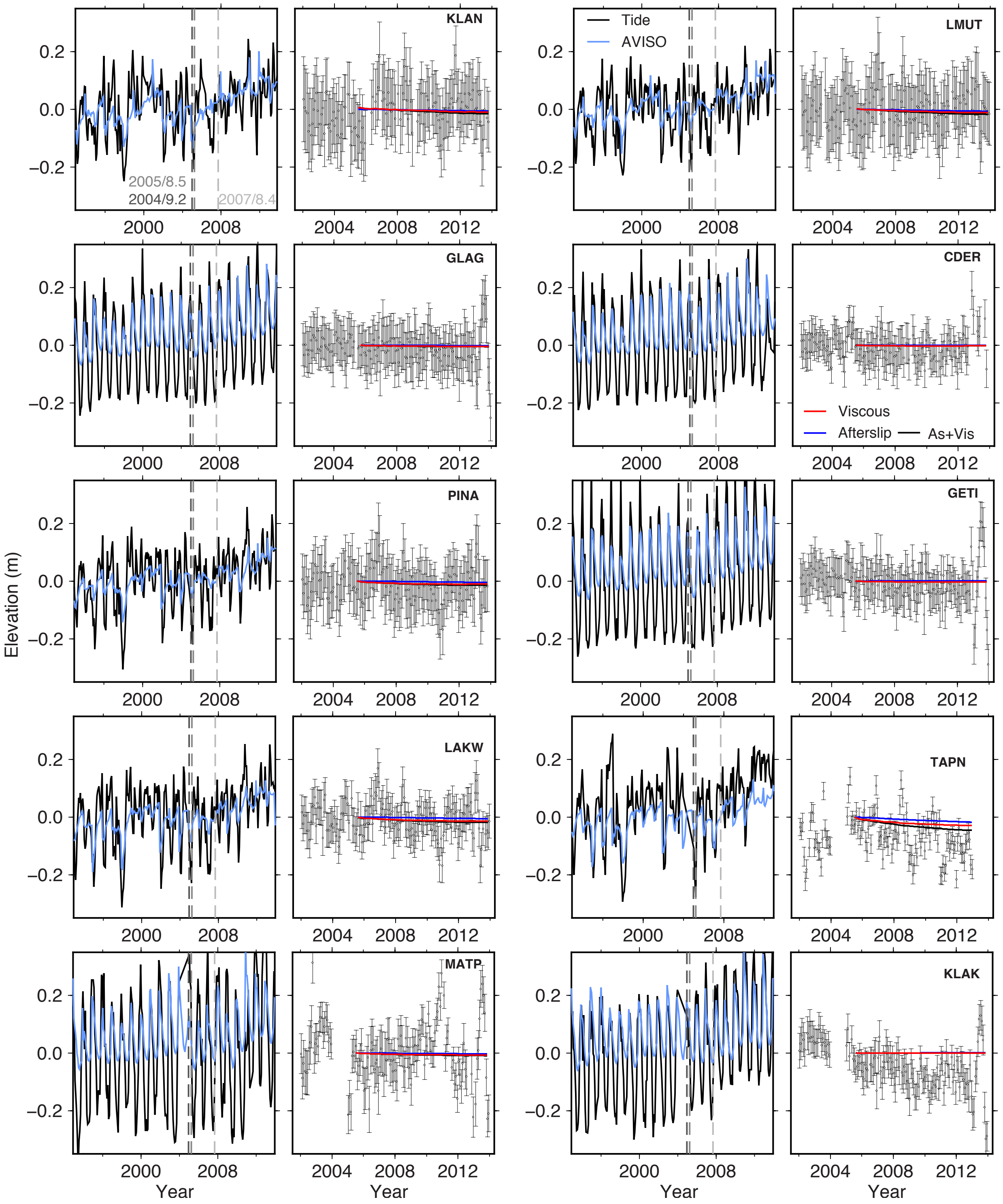


**Supplementary Figure. 5**: **Continued.**

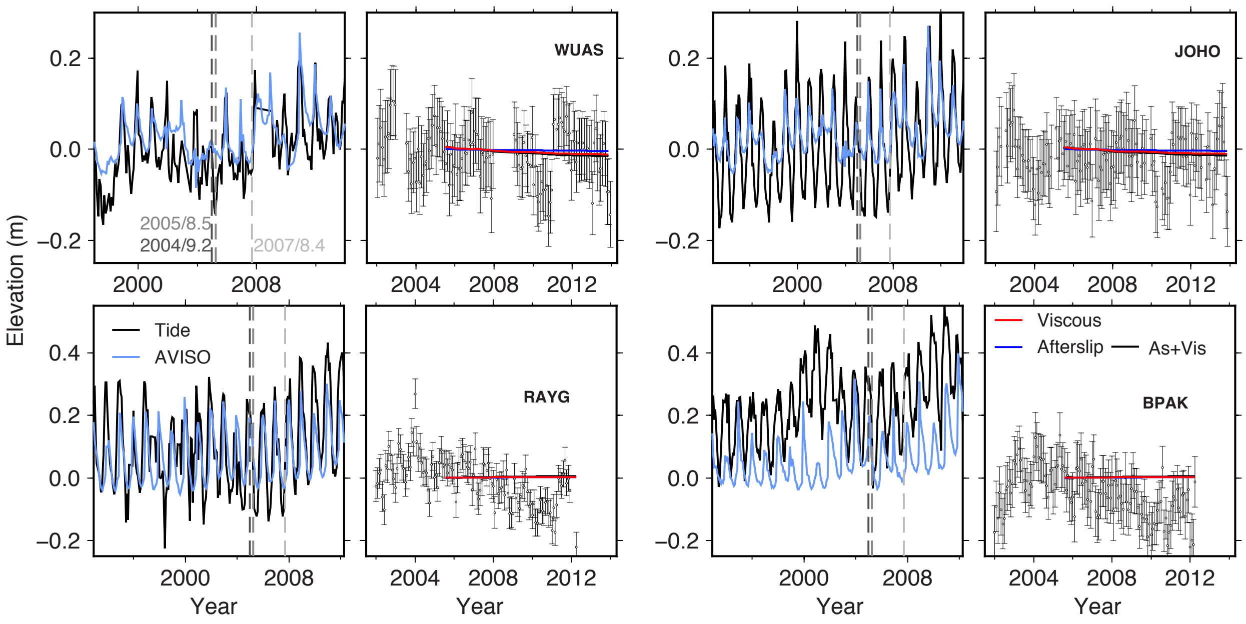


**Supplementary Figure 5. Continued.**

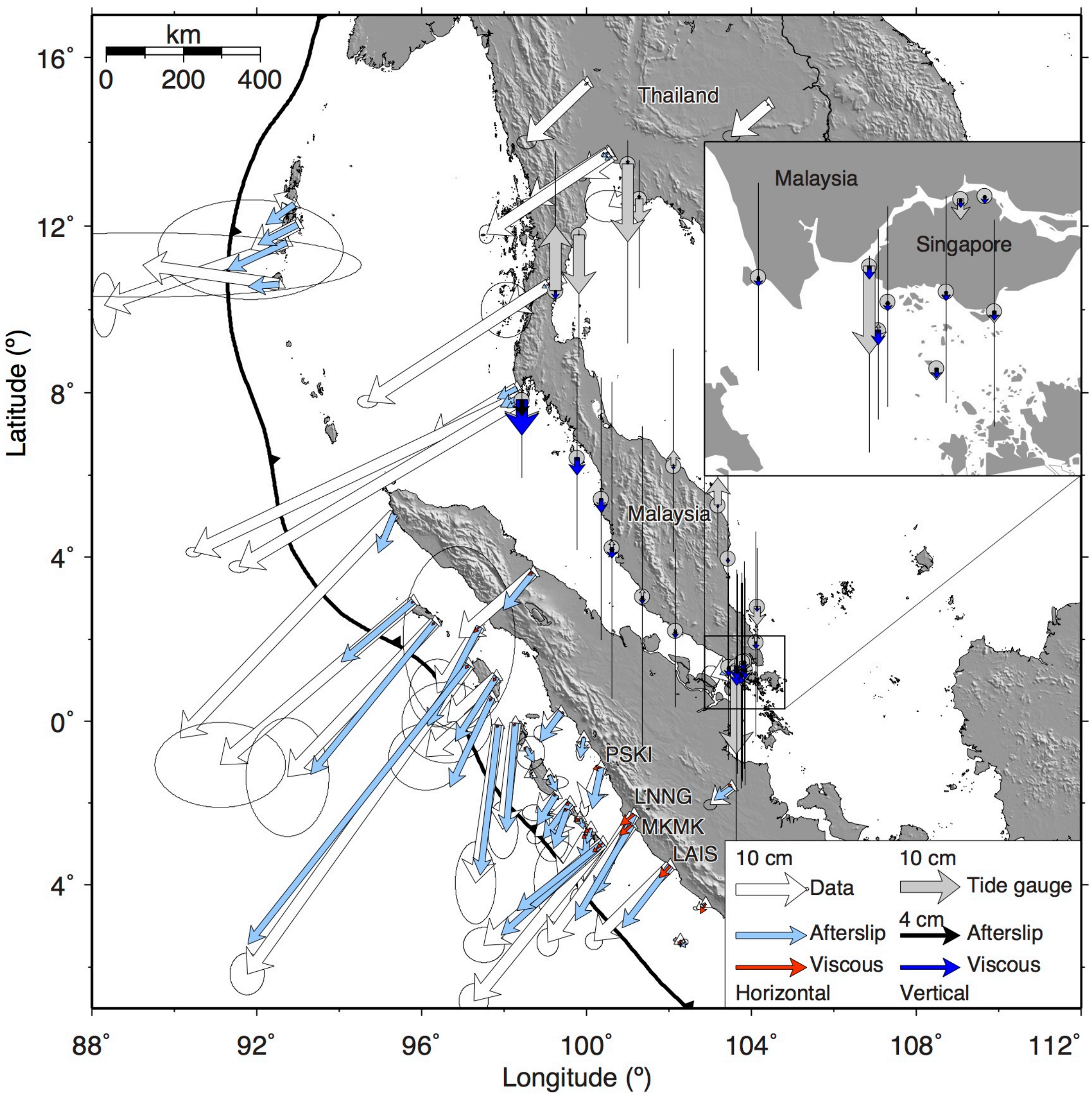


**Supplementary Figure 6. Our estimated cumulative displacements from mechanical decomposition of afterslip and viscoelastic flow at all geodetic stations over the initial postseismic phase (6 months after the earthquakes).** White vectors represent the cumulative horizontal displacements at GPS stations with 1σ error ellipses representing the 95% confidence intervals. Light blue vectors show the cumulative horizontal displacements due to afterslip on the megathrust. Red vectors indicate the cumulative horizontal displacements from viscoelastic flow in the mantle wedge. Grey vectors represent the vertical land displacements extracted from tide gauge measurement and Aviso altimetry data. Black and blue vectors indicate the predicted vertical displacements from afterslip and viscoelastic flow respectively. Due to the large variation of the tide gauge vertical displacements and its large uncertainties, different scales are used for the tide gauge vertical displacements from afterslip and viscoelastic flow respectively.

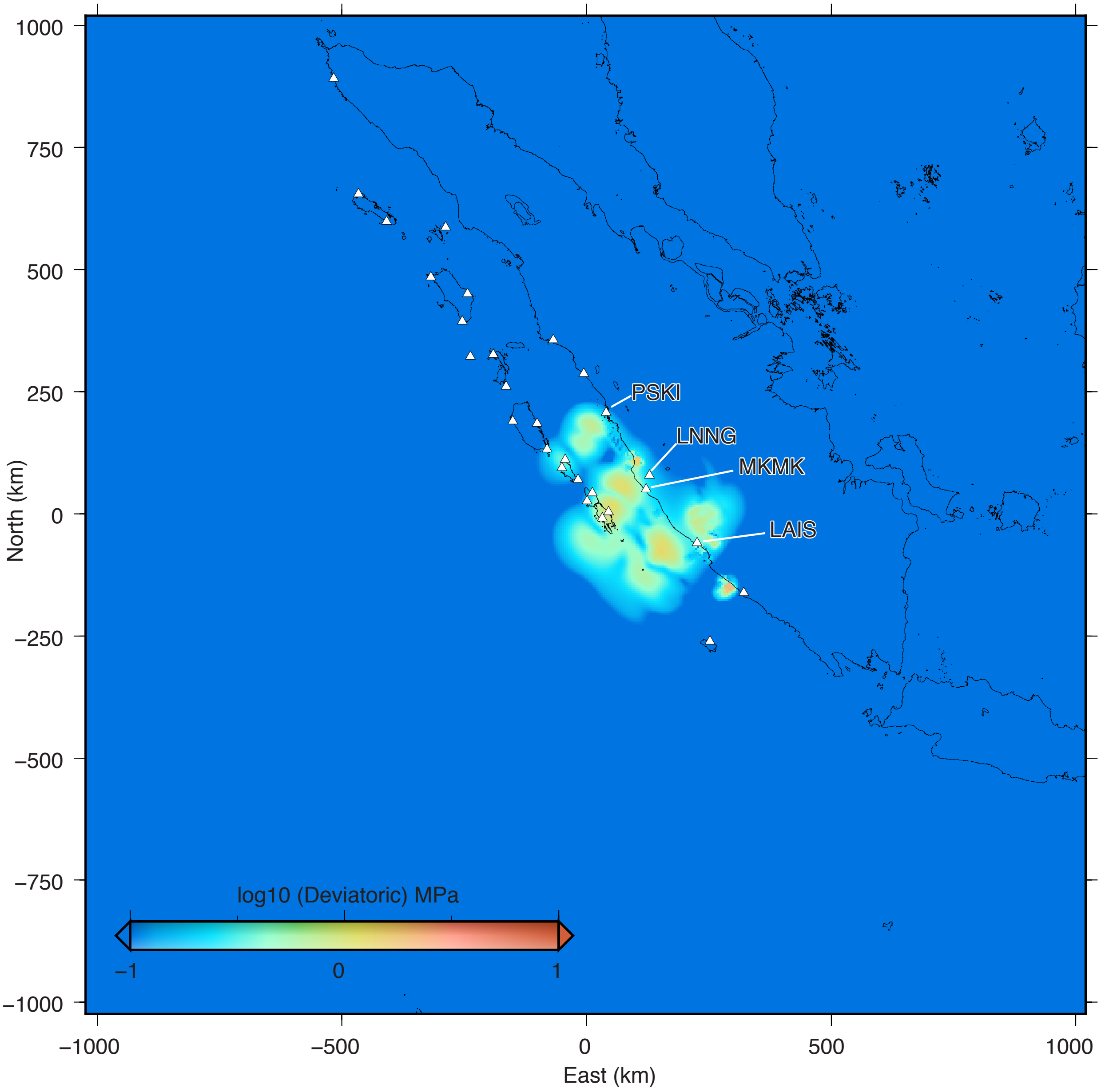


**Supplementary Figure 7. The second invariant of the coseismic stress tensor from the Mw 8.4 Bengkulu earthquake[1]. The colour bar is saturated.**

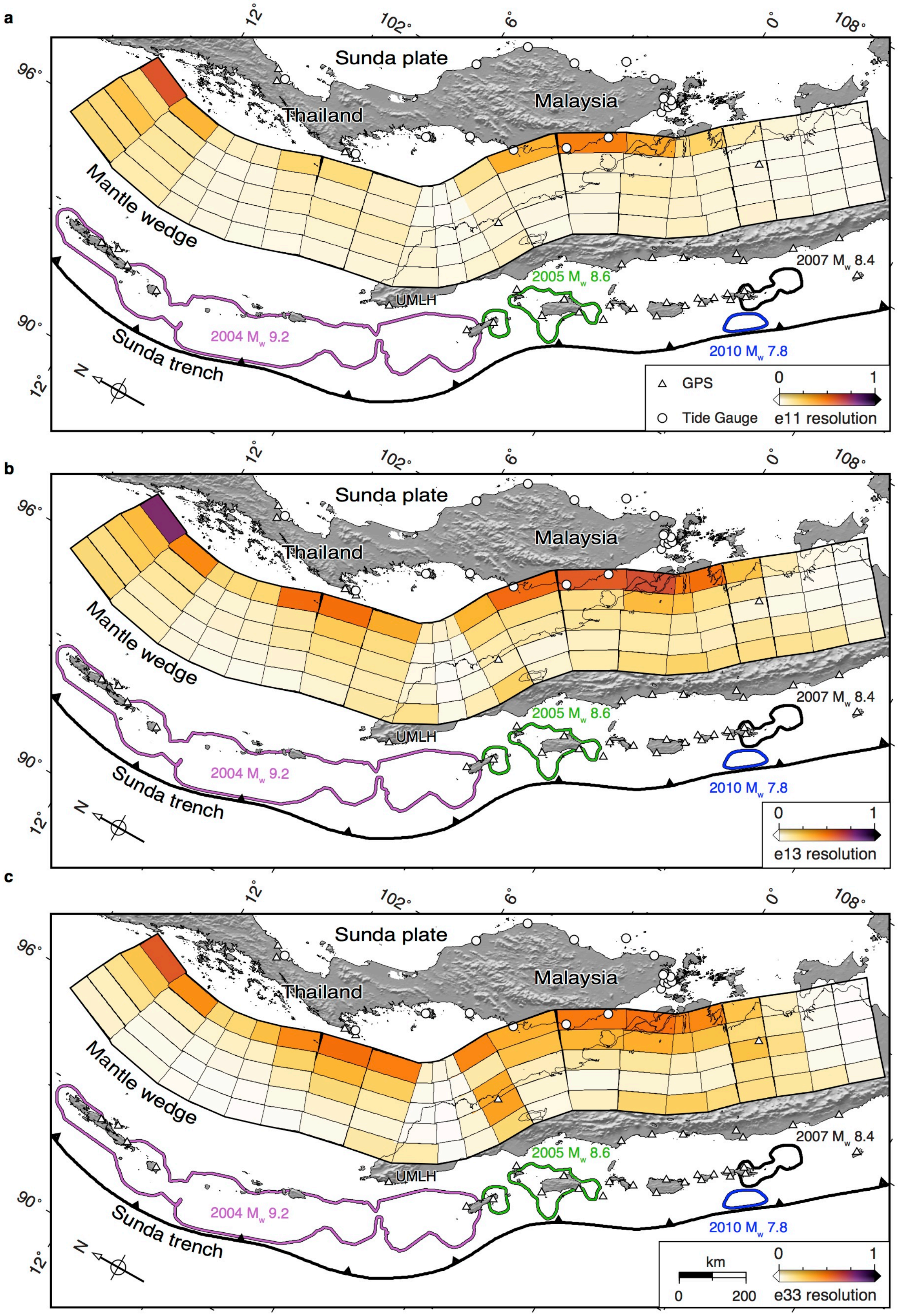


**Supplementary Figure 8. Resolution matrix for strain component e11, e13 and e33, which essentially govern the viscous shear motions in the mantle wedge.** We see an increasing resolution as approaching the far-field GPS and tide gauges.

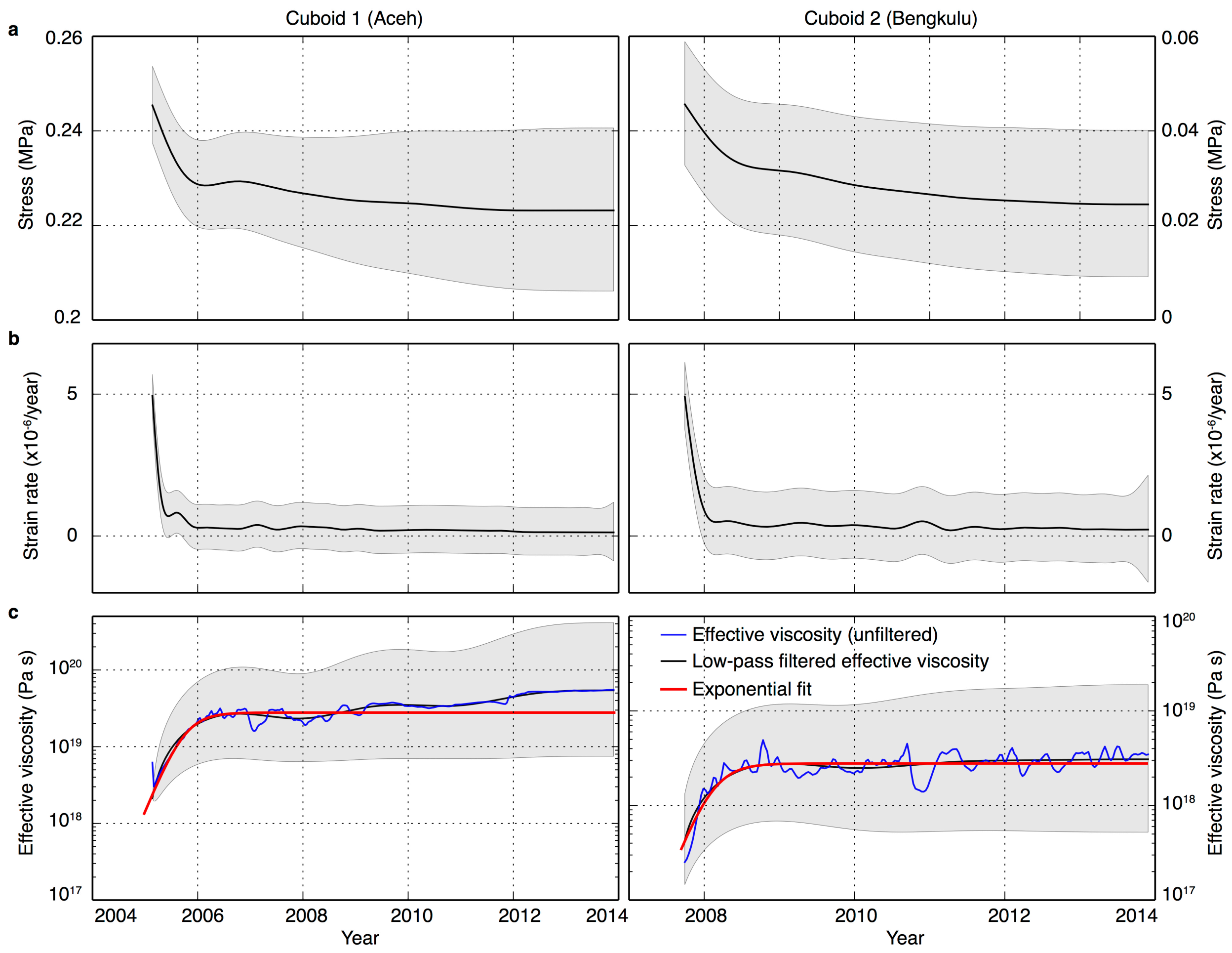


**Supplementary Figure 9. Time evolution of estimated stress, strain rates and effective viscosities for sample cuboids 1 and 2 from Fig. 6c.**

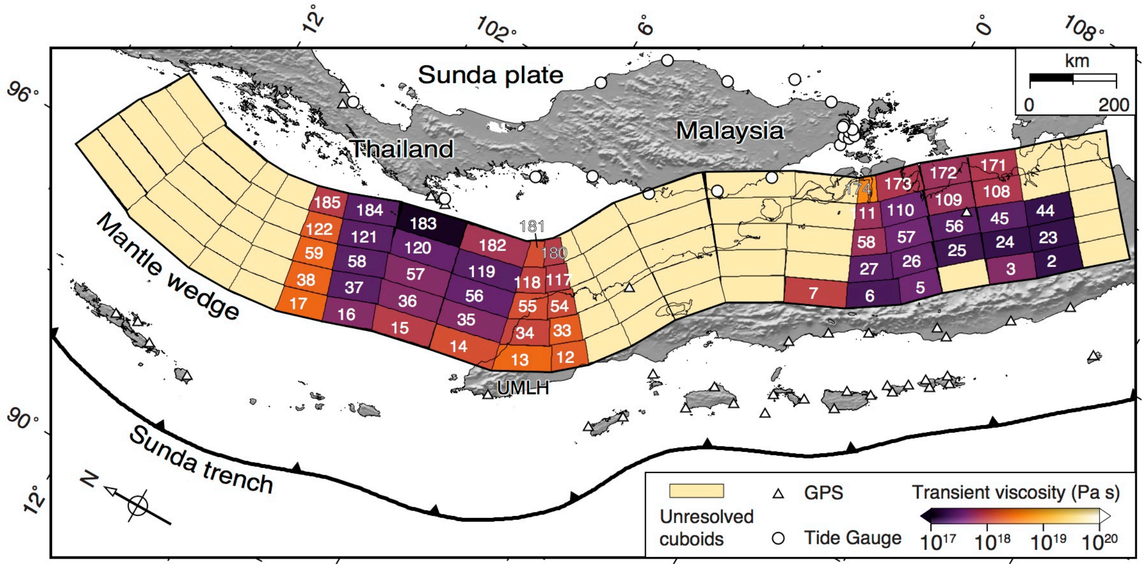


**Supplementary Figure 10. Index of the well-resolved cuboids that are listed out in Supplementary Table 1.** The background colour indicates the estimate transient viscosity (the same as Fig. 6a).

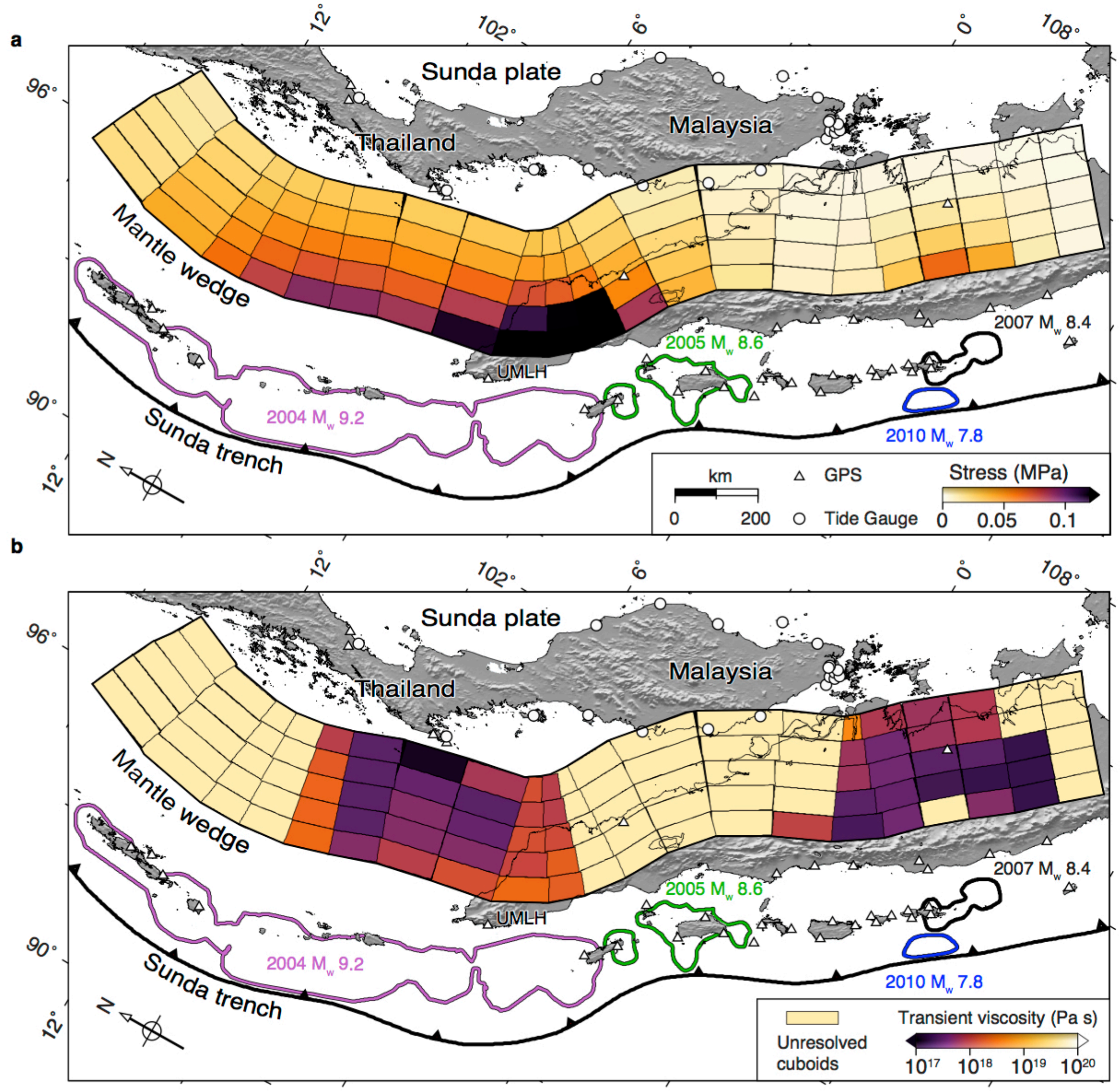


**Supplementary Figure 11. Spatial relationship of the second invariant coseismic stress tensor from the 2004 $M_w$ 9.2 Sumatra–Andaman[2], the 2005 $M_w$ 8.6 Nias–Simeulue[3], the 2007 $M_w$ 8.4 Bengkulu[1] earthquakes, and the inverted transient viscosities. a.** The second invariant coseismic stress tensor. Colours indicate the magnitude of the stress and is saturated. **b.** The inverted transient viscosity at resolved cuboids at the Sumatra-Andaman and the Bengkulu segments. The coseismic slip contours are the same as Fig. 1 & 6.

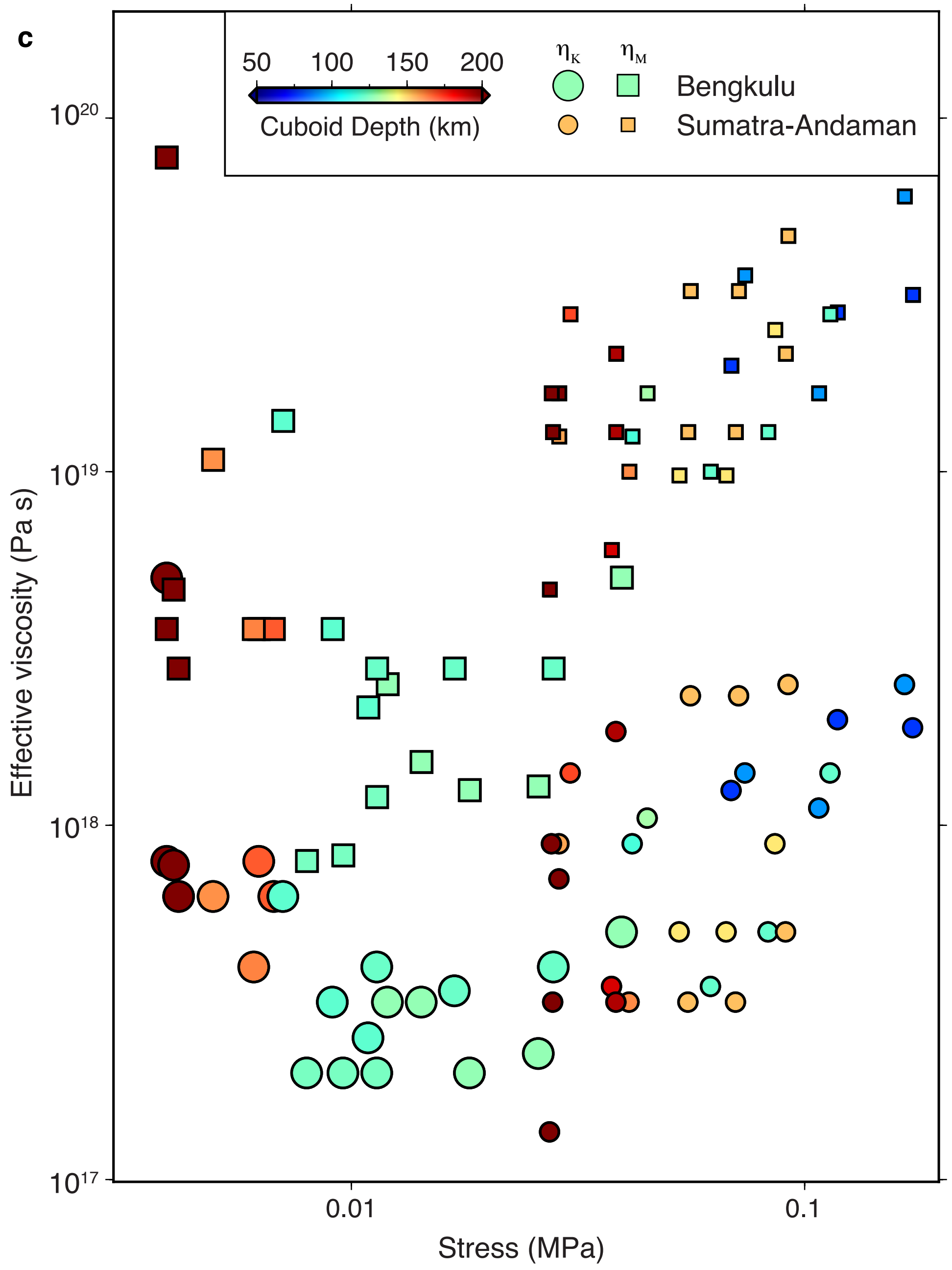


**Supplementary Figure 11. Continued. c. Relationship of the second invariant coseismic stress tensor, and the inverted transient and steady-state viscosities.** Different size and shape of symbols indicate transient and steady-state viscosities at resolved cuboids located at the Sumatra-Andaman and the Bengkulu segments. Colours show the depth of cuboids.

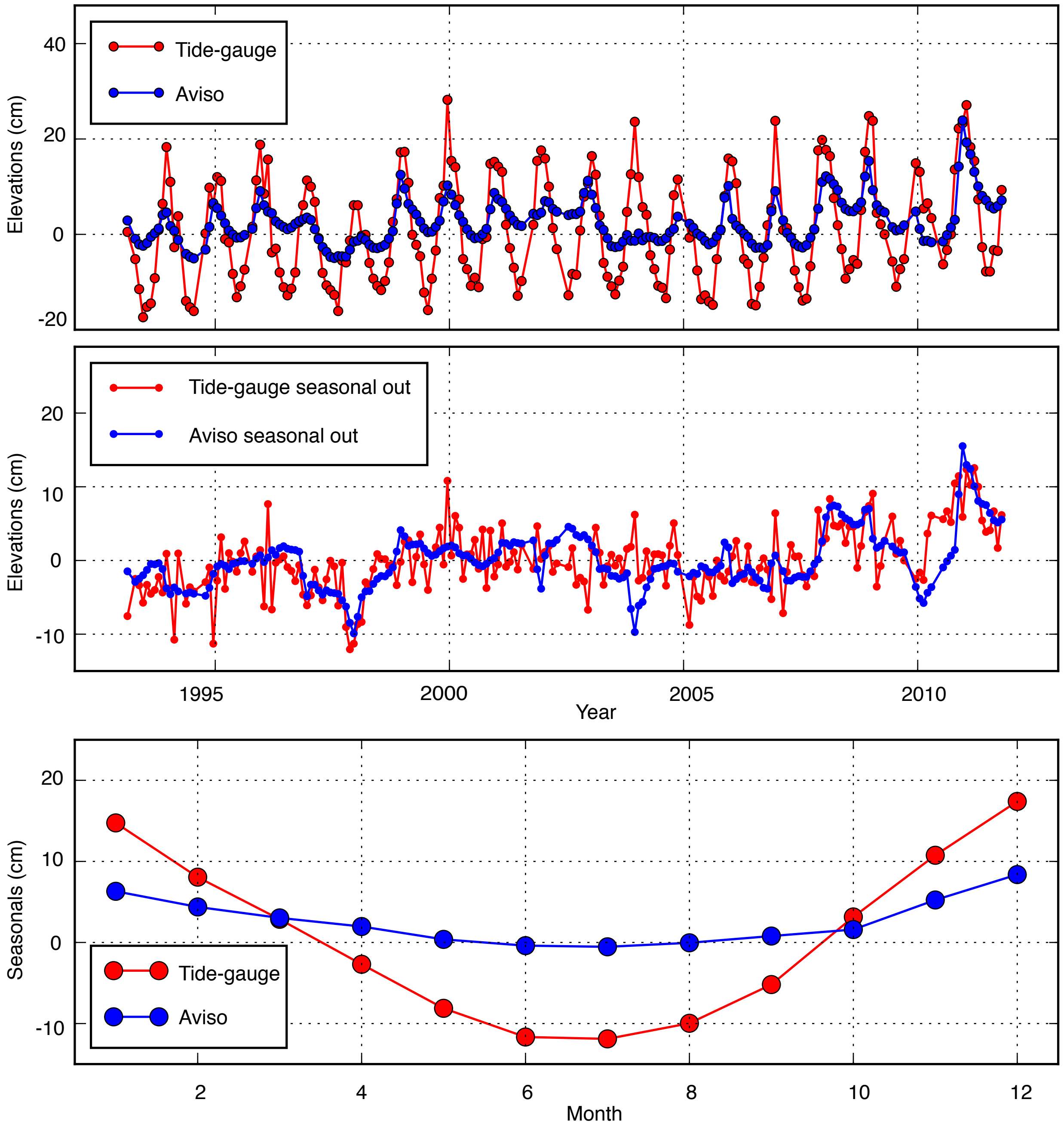


**Supplementary Figure 12. An example of the seasonal variations in both tide-gauge and Aviso time series.** We subtracted these average seasonal signals from the monthly average time series.

## Supplementary tables

**Supplementary Table 1. Published rheology model parameters and rheological parameter estimates of this study.** Published bi-viscous rheology model parameters (e.g., transient viscosity and steady-state viscosities and time scale of transient), and our estimates for the best-resolved cuboids shown in Supplementary Fig. 12.

| Cuboid Index | Transient viscosity (Pa s) | Steady state viscosity (Pa s) | Time scale of transient (yr) |
|---|---|---|---|
| 2 | $2.00 \times 10^{17}$ | $1.20 \times 10^{18}$ | 0.28 |
| 3 | $5.01 \times 10^{17}$ | $5.01 \times 10^{18}$ | 0.26 |
| 5 | $3.98 \times 10^{17}$ | $2.78 \times 10^{18}$ | 0.79 |
| 6 | $2.51 \times 10^{17}$ | $2.15 \times 10^{18}$ | 0.16 |
| 7 | $1.00 \times 10^{18}$ | $7.74 \times 10^{18}$ | 0.26 |
| 12 | $1.90 \times 10^{18}$ | $3.16 \times 10^{19}$ | 0.20 |
| 13 | $2.51 \times 10^{18}$ | $5.99 \times 10^{19}$ | 0.16 |
| 14 | $1.41 \times 10^{18}$ | $2.78 \times 10^{19}$ | 0.25 |
| 15 | $8.91 \times 10^{17}$ | $2.51 \times 10^{19}$ | 0.21 |
| 16 | $5.01 \times 10^{17}$ | $2.15 \times 10^{19}$ | 0.20 |
| 17 | $2.51 \times 10^{18}$ | $4.64 \times 10^{19}$ | 0.26 |
| 23 | $2.00 \times 10^{17}$ | $8.24 \times 10^{17}$ | 0.21 |
| 24 | $2.00 \times 10^{17}$ | $1.26 \times 10^{18}$ | 0.26 |
| 25 | $2.27 \times 10^{17}$ | $1.29 \times 10^{18}$ | 0.20 |
| 26 | $3.41 \times 10^{17}$ | $2.78 \times 10^{18}$ | 0.13 |
| 27 | $3.16 \times 10^{17}$ | $3.59 \times 10^{18}$ | 0.15 |
| 33 | $2.00 \times 10^{18}$ | $2.82 \times 10^{19}$ | 0.21 |
| 34 | $1.12 \times 10^{18}$ | $1.67 \times 10^{19}$ | 0.21 |
| 35 | $5.01 \times 10^{17}$ | $1.29 \times 10^{19}$ | 0.22 |
| 36 | $5.01 \times 10^{17}$ | $9.77 \times 10^{18}$ | 0.23 |
| 37 | $3.16 \times 10^{17}$ | $1.29 \times 10^{19}$ | 0.19 |
| 38 | $2.33 \times 10^{18}$ | $3.24 \times 10^{19}$ | 0.26 |
| 44 | $2.00 \times 10^{17}$ | $7.94 \times 10^{17}$ | 0.31 |
| 45 | $3.16 \times 10^{17}$ | $2.51 \times 10^{18}$ | 0.17 |
| 46 | $3.16 \times 10^{17}$ | $1.51 \times 10^{18}$ | 0.21 |
| 47 | $3.98 \times 10^{17}$ | $2.78 \times 10^{18}$ | 0.11 |
| 48 | $6.31 \times 10^{17}$ | $1.39 \times 10^{19}$ | 0.16 |
| 54 | $1.26 \times 10^{18}$ | $2.00 \times 10^{19}$ | 0.22 |
| 55 | $1.41 \times 10^{18}$ | $3.59 \times 10^{19}$ | 0.19 |
| 56 | $3.50 \times 10^{17}$ | $1.00 \times 10^{19}$ | 0.18 |
| 57 | $5.01 \times 10^{17}$ | $9.77 \times 10^{18}$ | 0.25 |
| 58 | $3.16 \times 10^{17}$ | $1.29 \times 10^{19}$ | 0.18 |
| 59 | $2.33 \times 10^{18}$ | $3.24 \times 10^{19}$ | 0.24 |
| 108 | $7.94 \times 10^{17}$ | $3.59 \times 10^{18}$ | 0.22 |
| 109 | $6.31 \times 10^{17}$ | $3.59 \times 10^{18}$ | 0.19 |
| 110 | $3.98 \times 10^{17}$ | $3.59 \times 10^{18}$ | 0.10 |
| 111 | $6.31 \times 10^{17}$ | $1.08 \times 10^{19}$ | 0.13 |
| 117 | $8.91 \times 10^{17}$ | $1.26 \times 10^{19}$ | 0.23 |

| | | | |
|---|---|---|---|
| 118 | 1.05 x$10^{18}$ | 1.67 x$10^{19}$ | 0.18 |
| 119 | 3.16 x$10^{17}$ | 1.00 x$10^{19}$ | 0.30 |
| 120 | 3.50 x$10^{17}$ | 5.99 x$10^{18}$ | 0.18 |
| 121 | 3.16 x$10^{17}$ | 1.29 x$10^{19}$ | 0.18 |
| 122 | 1.85 x$10^{18}$ | 2.15 x$10^{19}$ | 0.25 |
| 171 | 7.94 x$10^{17}$ | 3.59 x$10^{18}$ | 0.18 |
| 172 | 6.31 x$10^{17}$ | 2.78 x$10^{18}$ | 0.28 |
| 173 | 7.74 x$10^{17}$ | 4.64 x$10^{18}$ | 0.14 |
| 174 | 5.01 x$10^{18}$ | 7.74 x$10^{19}$ | 0.23 |
| 180 | 8.91 x$10^{17}$ | 1.26 x$10^{19}$ | 0.23 |
| 181 | 1.41 x$10^{18}$ | 2.78 x$10^{19}$ | 0.20 |
| 182 | 7.08 x$10^{17}$ | 1.67 x$10^{19}$ | 0.21 |
| 183 | 1.36 x$10^{17}$ | 4.64 x$10^{18}$ | 0.18 |
| 184 | 3.16 x$10^{17}$ | 1.29 x$10^{19}$ | 0.16 |
| 185 | 8.91 x$10^{17}$ | 1.67 x$10^{19}$ | 0.22 |
| Published models | Transient viscosity (Pa s) | Steady state viscosity (Pa s) | Time scale of transient (yr) |
| Ref. [4] | 5.0 x$10^{17}$ | 5.0 x$10^{18}$ | 0.22 |
| Ref. [5] | 4.0 x$10^{17}$ | 8.0 x$10^{18}$ | 0.18 |
| Ref. [6] | 5.0 x$10^{17}$ | 1.0 x$10^{19}$ | 0.22 |
| Ref. [7] | 1.0 x$10^{18}$ | 1.0 x$10^{19}$ | 1.30 |
| Ref. [8] | 5.0 x$10^{17}$ | 1.0 x$10^{19}$ | 0.24 |
| Ref. [9] | 1.0 x$10^{18}$ | 7.5 x$10^{18}$ | 0.47 |

## Supplementary References


1. Konca, A. O. *et al.* Partial rupture of a locked patch of the Sumatra megathrust during the 2007 earthquake sequence. *Nature* **456,** 631–635 (2008).
2. Chlieh, M. *et al.* Coseismic Slip and Afterslip of the Great Mw 9.15 Sumatra-Andaman Earthquake of 2004. *Bull. Seismol. Soc. Am.* **97,** S152–S173 (2007).
3. Konca,  a. O. *et al.* Rupture Kinematics of the 2005 Mw 8.6 Nias-Simeulue Earthquake from the Joint Inversion of Seismic and Geodetic Data. *Bull. Seismol. Soc. Am.* **97,** S307–S322 (2007).
4. Han, S.-C., Sauber, J., Luthcke, S. B., Ji, C. & Pollitz, F. F. Implications of postseismic gravity change following the great 2004 Sumatra-Andaman earthquake from the regional harmonic analysis of GRACE intersatellite tracking data. *J. Geophys. Res. Solid Earth* **113,** B11413 (2008).
5. Panet, I. *et al.* Upper mantle rheology from GRACE and GPS postseismic deformation after the 2004 Sumatra-Andaman earthquake. *Geochemistry, Geophys. Geosystems* **11,** Q06008 (2010).
6. Pollitz, F., Banerjee, P., Grijalva, K., Nagarajan, B. & Bürgmann, R. Effect of 3-D viscoelastic structure on post-seismic relaxation from the 2004 Mw 9.2 Sumatra earthquake. *Geophys. J. Int.* **173,** 189–204 (2008).

7. Hoechner, A., Sobolev, S. V, Einarsson, I. & Wang, R. Investigation on afterslip and steady state and transient rheology based on postseismic deformation and geoid change caused by the Sumatra 2004 earthquake. *Geochemistry, Geophys. Geosystems* **12,** Q07010 (2011).
8. Hu, Y. & Wang, K. Spherical-Earth finite element model of short-term postseismic deformation following the 2004 Sumatra earthquake. *J. Geophys. Res. Solid Earth* **117,** B05404 (2012).
9. Broerse, T., Riva, R., Simons, W., Govers, R. & Vermeersen, B. Postseismic GRACE and GPS observations indicate a rheology contrast above and below the Sumatra slab. *J. Geophys. Res. Solid Earth* **120,** 5343–5361 (2015).
