# Supplementary material for: Transient rheology of the Sumatran mantle wedge revealed by a decade of great earthquakes
Source: Nat Commun. 2018 Mar 8;9:995. doi: 10.1038/s41467-018-03298-6 (PMC5843651; doi:10.1038/s41467-018-03298-6)
Supplement: Supplementary file 3 — Description of Additional Supplementary Files [file 41467_2018_3298_MOESM3_ESM.pdf]

Description of Additional Supplementary Files

File Name: Supplementary Movie 1
Description: C-animation.mp4 shows the time evolution and spatial variation of afterslip on the megathrust and the second invariant of the anelastic strain tensor in finite-volume cuboids in the mantle wedge along the Sumatran subduction zone from 2005 to 2014.
